# Using Proteomics to Stage Alzheimer's Disease

**DOI:** 10.1002/alz70856_098759

**Published:** 2025-12-24

**Authors:** Erik C.B. Johnson

**Affiliations:** ^1^ Department of Neurology, Emory University School of Medicine, Atlanta, GA, USA; Goizueta Alzheimer's Disease Research Center, Emory University, Atlanta, GA, USA

## Abstract

**Background:**

Alzheimer's disease (AD) and its associated neuropathology evolves over decades. Although biomarkers of amyloid‐β (Aβ) and tau pathology have been instrumental in defining different AD stages and trajectories of disease evolution over this time course, the sequence of neuropathological changes associated with AD other than Aβ and tau are less well understood.

**Method:**

We used targeted and discovery proteomics in cerebrospinal fluid (CSF) from control, preclinical, and clinical late‐onset sporadic and autosomal dominant Alzheimer's disease (LOAD and ADAD) to assess changes in CSF proteins linked to different brain pathologies associated with AD. The timing of these protein changes were benchmarked to changes in Aβ, tau, and cognitive function in ADAD. In LOAD, cross‐sectional data were used to infer possible disease stages using unbiased clustering approaches.

**Result:**

In ADAD, targeted proteomics revealed a temporal progression of pathologic changes starting with Aβ deposition associated with changes in extracellular matrix proteins, followed sequentially by changes in synaptic proteins, glycolytic metabolism and stress response proteins, white matter/axonal integrity, immune activation and cognitive decline, metabolic failure, and cortical atrophy. In LOAD, changes in multiple pathologies assessed by discovery proteomics were observed to occur in people classified as either AD or control based on Aβ and tau levels, but the temporal progression of pathological changes in LOAD was less clear.

**Conclusion:**

Biofluid proteomics can reveal multiple brain‐based pathologies that evolve over the course of AD and holds promise for staging the disease. Additional proteomic studies in longitudinal cohorts are required to better define staging in LOAD.

